# Supplementary material for: Chromothripsis during telomere crisis is independent of NHEJ, and consistent with a replicative origin
Source: Genome Res. 2019 May;29(5):737–49. doi: 10.1101/gr.240705.118 (PMC6499312; doi:10.1101/gr.240705.118)
Supplement: Supplemental Material [file supp_gr.240705.118_Supplemental_file_1.zip › contigs/annotated_contigs/DB93/contig.2.DB93_length_265_mean_cov_6.09433962264.docx]

**DB93_length_265_mean_cov_6.09433962264**

```
AGATTTGGATAATAATAGTATAACTGTTAAACAGAGATGCAAAAGGAAAATAGAAGCAACTGGAGTGATACAATCTTGTGCCAAAGAGG
 >chr8:17867165-17867272 + E=4e-53
TAAATAACGTTCATTTT|G|GTTCAAGTACTTAGTAAAGGCTAGTGCTCAAATATTTCCGCTTGTTCTCTTGCACCTTTACCACTGCCA
                  >chr17:52470131-52470290 + E=1e-84
TGAGAACACGACCAGGATGGCCTGATGAATATGAAAAATGCATGGGTCAGTGGCCCCTGTTGCTCCAGCGAACAGCCAGCCAAATGCCA
```
